# A burden of rare variants in *BMPR2* and *KCNK3* contributes to a risk of familial pulmonary arterial hypertension

**DOI:** 10.1186/s12890-017-0400-z

**Published:** 2017-04-07

**Authors:** Koichiro Higasa, Aiko Ogawa, Chikashi Terao, Masakazu Shimizu, Shinji Kosugi, Ryo Yamada, Hiroshi Date, Hiromi Matsubara, Fumihiko Matsuda

**Affiliations:** 1 Human Disease Genomics, Center for Genomic Medicine, Kyoto University Graduate School of Medicine, Kawara-cho 53, Shogoin, Sakyo-ku, Kyoto, 606-8507 Japan; 2 Department of Clinical Science, National Hospital Organization Okayama Medical Center, Okayama, Japan; 3 Department of Medical Ethics/Medical Genetics, Graduate School of Medicine, Kyoto University, Kyoto, Japan; 4 Statistical Genetics, Center for Genomic Medicine, Kyoto University Graduate School of Medicine, Kyoto, Japan; 5 Department of Thoracic Surgery, Graduate School of Medicine, Kyoto University, Kyoto, Japan

**Keywords:** Clinical genetic testing, Gene-based association study, Next generation sequencing, Pulmonary arterial hypertension

## Abstract

**Background:**

Pulmonary arterial hypertension (PAH) is a severe lung disease with only few effective treatments available. Familial cases of PAH are usually recognized as an autosomal dominant disease, but incomplete penetrance of the disease makes it difficult to identify pathogenic variants in accordance with a Mendelian pattern of inheritance.

**Methods:**

To elucidate the complex genetic basis of PAH, we obtained whole exome- or genome-sequencing data of 17 subjects from 9 families with heritable PAH and applied gene-based association analysis with 9 index patients and 300 PAH-free controls.

**Results:**

A burden of rare variants in *BMPR2* significantly contributed to the risk of the disease (*p* = 6.0 × 10^−8^). Eight of nine families carried four previously reported single nucleotide variants and four novel insertion/deletion variants in the gene. One of the novel variants was a large 6.5 kilobase-deletion. In the remaining one family, the patient carried a pathogenic variant in a member of potassium channels, *KCNK3,* which was the first replicative finding of channelopathy in an Asian population.

**Conclusions:**

The variety of rare pathogenic variants suggests that gene-based association analysis using genome-wide sequencing data from increased number of samples is essential to tracing the genetic heterogeneity and developing an appropriate panel for genetic testing.

**Electronic supplementary material:**

The online version of this article (doi:10.1186/s12890-017-0400-z) contains supplementary material, which is available to authorized users.

## Background

Pulmonary arterial hypertension (PAH, MIM #178600) is a rare vascular lung disease presenting increased pulmonary vascular resistance and elevation of mean pulmonary arterial pressure, leading to a grave prognosis of right heart failure without treatment. While survival rates are increasing with a number of recently developed treatments such as epoprostenol, the optimal care of patients with these therapies is unclear due to phenotypic variations and genetic backgrounds.

Despite the complex etiology of PAH due to incomplete penetrance and genetic heterogeneity, multiple genes that cause PAH have been discovered during the last few decades. *BMPR2* variants have been identified in not only >70% of heritable PAH (HPAH), but also 10–40% of idiopathic PAH (IPAH) [1–6]. Variants of other genes including *KCNK3*, *ACVRL1*, *ENG*, *CAV1*, and the *SMAD* family are also rare causes of PAH [7–11]. Furthermore, common variants in *CBLN2* and *KCNA5* were also reported to be associated with the risk of PAH in European Caucasians [12, 13].

Although identification of individuals who carry genetic variants that increase the risk of developing PAH offers an opportunity for earlier diagnosis and finding a therapeutic strategy, the majority of previous studies was only focused on the protein coding regions of the most frequently mutated gene *BMPR2* with the use of conventional methods such as Sanger sequencing [6, 14]. Thus, for the patients who have no mutation in *BMPR2*, i.e., around 30% of HPAH and 60–90% of IPAH, another approach, which is practical for multiple genes, is necessary. Considering that current state-of-the-art sequencing technologies allow us to access exome- and genome-wide variants with reasonable costs, unbiased screening of pathogenic variants is beneficial to continue expanding the genetic diagnosis catalogue. However, conventional segregation-based approaches, e.g., linkage analysis, do not have enough power to pinpoint the true pathogenic variants from these genome-wide candidate variants without functional evaluations, especially for diseases with phenotypic and genetic heterogeneity. In this study, applying a gene-based association test, we statistically evaluate the significance of rare variant enrichment in genes responsible for PAH. The strategy we employed here would be useful to unbiasedly elucidate the pathogenicity of multiple rare variants arising from independent founder events in Mendelian diseases as well as common diseases.

## Methods

### Subjects

**Fig. 1:**
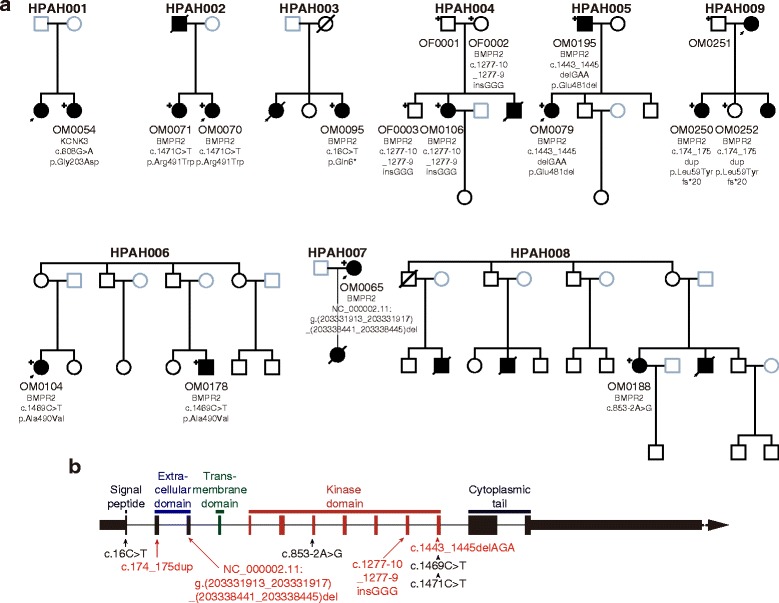
Segregation of the pathogenic variants identified in 9 familial PAH. **a** Eight families carried *BMPR2* variants and one family carried a *KCNK3* variant. Nucleotide and amino acid changes for *BMPR2* and *KCNK3* are described on NM_001204.6 and NM_002246.2, respectively. Index patients of each family are pointed with arrows. The subjects whose DNAs were available are indicated in plus signs. **b** All the possible pathogenic variants discovered in the eight PAH families were located before or in the kinase domain. Four previously reported and four novel variants were indicated with black and red letters, respectively

We consecutively enrolled five families with PAH and four individual cases with a family history of PAH to this study (Fig. 1). The patients have been diagnosed in the National Hospital Organization Okayama Medical Center between 1996 and 2014. All subjects who participated in our study were approved by the Institutional Review Board of our institutes in which donors gave written informed consent in accordance with institutional and national guidelines.

### Next-generation sequencing and data analysis

To understand comprehensive genetic background of these 9 PAH families, we applied whole exome- or genome-sequencing to 12 patients and 5 healthy family members whose DNA were available (Fig. 1 and Additional file 1: Table S1). For the exome sequencing, DNA fragments were enriched by SureSelectXT Human All Exon v4 + UTR (Agilent Technologies, Santa Clara, CA, USA) and then applied to SOLiD™ 5500XL sequencer (Thermo Fisher Scientific inc., Waltham, MA, USA). The whole genome sequencing was conducted with the Illumina HiSeq X sequencer (Illumina Inc., San Diego, CA, USA). After aligning the sequence reads onto the reference genome (NCBI Build 37) using the Burrows-Wheeler Aligner [15], downstream processes including the duplication removal, the recalibration of base quality values, the local realignment, the variant call, and the variant quality score recalibration were analyzed using GATK [16]. The variants were called with an exome sequencing data set of 300 control samples obtained from the Human Genome Variation Database (accession ID: HGV0000004) [17]. The resulting VCF file has been deposited on the same database under accession HGV0000005.

### Quality control for association study

After removing variants that were assigned as low quality by the GATK VariantRecalibrator, additional filters were applied to extract high quality variants such as low call rate (<0.9), excessive strand bias (FS > 50), haplotype score (> 5), deviation from Hardy-Weinberg equilibrium (InbreedingCoeff > 0.3), mapping quality of the reads (MQ < 35), excess of zero mapping quality (MQ0 > 100), bias of mapping quality between reference and alternative alleles (MQRankSum < 13), coverage over sample (DP/sample < 10), positional bias of the reads (ReadPosRankSum > 5), quality over depth (QD < 8), and low LOD score (VQSLOD < 0). For the gene-based association analysis, we selected likely protein damaging variants (premature termination, splice site, missense, and indels on exons) to perform the Variable Threshold (VT) test [18] implemented in Variant Association Tools [19].

### Annotation and screening of pathogenic variants

All identified variants were annotated using ANNOVAR [20]. Candidate pathogenic variants were screened according to the registrations and frequencies of the variants in the public databases: dbSNP (Build 147) [21], The 1000 Genomes (November 2010 data release) [22], The 10Gen Data Set (version 1.04) [23], NHLBI GO Exome Sequencing Project (ESP6500SI) [24], the Human Genetic Variation Database [17] or ClinVar [25]. For missense variants, PolyPhen-2 [26] and Mutation Taster [27], LRT [28] and PhyloP [29] score were obtained from the dbNSFP database [30]. Damaging effects of splice site variants were evaluated with MaxEntScan [31] and Human Splicing Finder [32].

## Results

### Gene-based association study

**Fig. 2:**
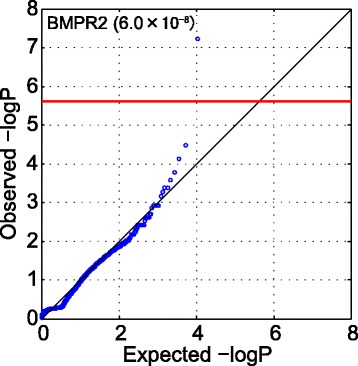
Gene-based association analysis. Quantile-quantile plot for VT test of the nine cases and 300 controls are shown. *BMPR2* was the only gene that surpassed the genome-wide significance threshold for 21,000 genes

To identify genes responsible for the pathogenesis of PAH, we applied a gene-based association test (Variable Threshold test [18]) to the 60,367 damaging variants extracted from the nine PAH patients and the 300 control samples. These variants were located within 10,744 gene regions. Despite the small sample size, the burden of association between *BMPR2* and PAH was highly significant (*p* = 6.0 × 10^−8^) compared to the genome-wide significance threshold (*p* < 2.4 × 10^−6^) after Bonferroni correction for approximately 21,000 genes (Fig. 2). No other gene was found beyond the threshold.

### Identifying the pathogenic variants

The spectrum of rare variants found in *BMPR2* is summarized in Table 1. Of the nine families, four carried previously reported single nucleotide pathogenic variants (2 missenses, 1 nonsense, and 1 splice site) [1, 4, 6] and four carried novel insertions/deletions (indels) in this gene (88.9%). One of the novel indels was a large deletion of 6.5 kilobases in length by which one allele lacks the entire region of exon 3 (Additional file 2: Figure S1). Two variants are suspected to be pathogenic although showing incomplete penetrance, since clinically unaffected subjects in the families harbored the same variants found in the patients (Table 1). There were no *BMPR2* variants observed in the remaining one of nine families, but we identified one heterozygous missense variant (p.Gly203Asp) in *KCNK3* by screening the previously reported pathogenic variants [25]. This variant was shown to disrupt the ion-channel function by patch-clump analysis [11]. None of the pathogenic variants we identified was observed in the 300 control samples or in the public database for the Japanese population [17]. Of these, all three missense variants were occurred at highly conserved nucleotides among vertebrates and were assumed to be damaging to the protein function by at least three *in silico* prediction programs [26–29] (Table 1).

**Table 1:** Summary of pathogenic variants and clinical features

Family ID	Sample ID	Gender	Diagnosis	Age at diagnosis (y.o.)	mPAP^a^ (mmHg)	CI^b^ (L/min/m^2^)	PVR^c^ (dyne·sec/cm^5^)	Chromosome	Position	Gene	Exon	Nucleotide change^d^	Amino acid change^d^	rs ID	Polyphen	Mutation taster	LRT	PhyloP	MaxEnt scan	Human splice finder	Quality score^e^	Reference^f^
1	OM0054	F	HPAH	23	81	2.3	1623	2	26,950,859	KCNK3	2	c.608G > A	p.Gly203Asp	-	1.000	1.000	0.000	2.407	N.A.	N.A.	179.78	Ma et al.[11], HPAH
2	OM0070	F	HPAH	23	55	1.8	1446	2	203,417,496	BMPR2	11	c.1471C > T	p.Arg491Trp	rs137852746	1.000	1.000	0.000	2.582	N.A.	N.A.	854.18	Deng et al.[1], H/IPAH
2	OM0071	F	HPAH	27	59	1.8	1371	2	203,417,496	BMPR2	11	c.1471C > T	p.Arg491Trp	rs137852746	1.000	1.000	0.000	2.582	N.A.	N.A.	854.18	Deng et al.[1], H/IPAH
3	OM0095	F	HPAH	30	62	2.7	730	2	203,242,213	BMPR2	1	c.16C > T	p.Gln6*	-	-	N.A.	N.A.	N.A.	N.A.	N.A.	772.78	Momose et al.[6], HPAH
4	OF0001	M	-	-	-			-	-	-	-	-	-	-	-	-	-	-	-	-	-	-
4	OF0002	F	-	-	-			2	203,407,024	BMPR2	10	c.1277–10_1277–9insGGG	splicing	-	-	N.A.	N.A.	N.A.	235.06	216.94	1932.84	-
4	OF0003	M	-	-	-			2	203,407,024	BMPR2	10	c.1277–10_1277–9insGGG	splicing	-	-	N.A.	N.A.	N.A.	235.06	216.94	1932.84	-
4	OM0106	F	HPAH	27	47	3.0	609	2	203,407,024	BMPR2	10	c.1277–10_1277–9insGGG	splicing	-	-	N.A.	N.A.	N.A.	235.06	216.94	1932.84	-
5	OM0079	F	HPAH	29	50	1.6	1556	2	203,417,465	BMPR2	11	c.1443_1445delAGA	p.Glu481del	-	-	N.A.	N.A.	N.A.	N.A.	N.A.	600.26	-
5	OM0195	M	HPAH	61	67	1.3	2234	2	203,417,465	BMPR2	11	c.1443_1445delAGA	p.Glu481del	-	-	N.A.	N.A.	N.A.	N.A.	N.A.	600.26	-
6	OM0104	F	HPAH	23	67	2.1	1257	2	203,417,494	BMPR2	11	c.1469C > T	p.Ala490Val	-	1.000	1.000	0.000	2.582	N.A.	N.A.	598.10	Machado et al.[4], IPAH
6	OM0178	M	HPAH	34	63	3.3	522	2	203,417,494	BMPR2	11	c.1469C > T	p.Ala490Val	-	1.000	1.000	0.000	2.582	N.A.	N.A.	598.10	Machado et al.[4], IPAH
7	OM0065	F	HPAH	26	47	3.5	636	2	203,331,443 ~ 203,338,403	BMPR2	3	NC_000002.11:g.(203331913_203331917)_(203338441_203338445)del	exon 3 deletion	-	-	N.A.	N.A.	N.A.	N.A.	N.A.	N.A.	-
8	OM0188	F	HPAH	36	70	1.9	1720	2	203,384,808	BMPR2	7	c.853-2A > G	splicing			N.A.	N.A.	N.A.	−85.96	−34.14	341.83	Machado et al.[4], IPAH
9	OM0250	F	HPAH	27	50	2.6	720	2	203,329,626	BMPR2	2	c.174_175dup	p.Leu59Tyrfs*20	-	-	N.A.	N.A.	N.A.	N.A.	N.A.	940.89	-
9	OM0251	M	-	-	-			-	-	-	-	-	-	-	-	-	-	-	-	-	-	-
9	OM0252	F	-	-	-			2	203,329,626	BMPR2	2	c.174_175dup	p.Leu59Tyrfs*20	-	-	N.A.	N.A.	N.A.	N.A.	N.A.	940.89	-

^a^mPAP: mean pulmonary artery pressure

^b^CI: cardiac index

^c^PVR: pulmonary vascular resistance

^d^Nucleotide and amino acid changes for BMPR2 and KCNK3 are described on NM_001204.6 or NM_002246.2, respectively, according to the Human Genome Variation Society nomenclature

^e^Quality score from GATK

^f^HPAH, IPAH, H/IPAH: The same pathogenic variants were found either in one of HPAH, IPAH or both HPAH and IPAH patient(s) in the previous reports

## Discussion

To our knowledge, this is the first report of gene-based genome-wide association analysis of HPAH. A burden of rare variants in *BMPR2* significantly contributes to risk of the disease (*p* = 6.0 × 10^−8^). The approach robustly detected the gene having a large effect on the pathogenesis of PAH, despite the genetic heterogeneity. Eight probands in the nine families harbored possible pathogenic variants in *BMPR2*. Half of these variants were novel indels. One of the novel indels was a large 6.5 kilobase deletion spanning the entire region of exon 3. Another novel indel was a three base insertion (NM_001204.6:c.1277-10_1277-9insGGG) in intron 9 (Additional file 3: Figure S2). Although we could not dismiss that this insertion has no responsibility to the disease pathogenicity, a potential creation of a new splice acceptor site by this insertion was strongly suggested from the multiple splice site prediction tools (Table 1, Additional file 4: Figure S3 and Additional file 5: Figure S4) [31, 32]. The remaining one patient harbored a missense variant in *KCNK3*, which was the first replicative finding of channelopathy in Japanese population. Among the nine families, all variants identified here were mutually exclusive, suggesting that the variants have originated from independent genetic founder events.

Patients who suffered from chronic lung diseases such as chronic obstructive pulmonary disease (COPD) and pulmonary fibrosis are prone to pulmonary hypertension (PH) development. They are categorized as Group 3 in the latest guidelines [33]. Most patients with COPD develop mild PH but 3–5% of them show a further rise in mean pulmonary arterial pressure >35 mmHg. It is unknown how “severe PH-COPD”, formerly known as “out-of-proportion PH” is induced. Furthermore, the PAH-approved drugs are yet to be approved for the patients with Group 3 PH. In this study, a male patient (OM0195 in HPAH005) had been treated at another hospital for COPD. He was later diagnosed with PH and referred to our hospital. This patient could be categorized as “severe PH-COPD”, if none of his family members developed PAH. Since we had treated his daughter for IPAH at our hospital, we clinically diagnosed them as HPAH. Genetic testing revealed an in-frame-deletion (c.1443_1445delGAA) in *BMPR2* in both patients. Underlying genetic predisposition might be one of the reasons for developing “severe PH-COPD”. Given our finding and a similarity of morphological appearance of vascular lesions between Group 1 and “severe PH-COPD” patients [33], PAH-approved drug treatments tailored to genetic diagnosis could well be a therapeutic strategy for such patients.

## Conclusions

According to the genetic testing registry at the National Institutes of Health, the available panels for clinical genetic testing for PAH do not include *KCNK3* and the detection methods are limited. Considering that pathogenic variants could occur within or spanning non-coding regions with a variety of sizes, the sequencing of the entire region of candidate genes is recommended to further understand the genetic factors relevant to PAH. This strategy will be essential for improving genetic diagnosis and counseling for PAH.

## Additional files

Additional file 1: Table S1. Summary of mapping statistics. (PDF 22 kb)

Additional file 2: Figure S1. Large deletion found in the patient in family 8. (a) The large deletion was supported by depth of coverage and discordant paired-end reads. (b) The length of the deletion was approximately 6.5 kilobases and spanned the entire region of exon 3. Breakpoints of the large heterozygous deletion were located in the Alu repeat sequences, which were supported by read depths and paired-end reads spanning the long region. (PDF 529 kb)

Additional file 3: Figure S2. Possible pathogenic variants found in the intron of BMPR2 in family 4. A rare three base insertion (GGG) at 10 bases upstream from exon 10 creates identical sequences around the canonical splicing site (ACAGGG). By the insertion, an out-of-frame protein could be translated due to the new splice acceptor site. (PDF 411 kb)

Additional file 4: Figure S3. Creation of the new splice acceptor site by the three base insertion (NM_001204.6:c.1277-10_1277-9insGGG) predicted from *in silico* programs. (PDF 218 kb)

Additional file 5: Figure S4. Disruption of wild type acceptor site by the single nucleotide variants (c.853-2A > G) predicted from *in silico* programs. (PDF 184 kb)

## Abbreviations

COPD: Chronic obstructive pulmonary disease
HPAH: Heritable PAH
IPAH: Idiopathic PAH
PAH: Pulmonary arterial hypertension
