# Supplementary material for: A burden of rare variants in BMPR2 and KCNK3 contributes to a risk of familial pulmonary arterial hypertension
Source: BMC Pulm Med. 2017 Apr 7;17:57. doi: 10.1186/s12890-017-0400-z (PMC5383973; doi:10.1186/s12890-017-0400-z)
Supplement: Supplementary file 1 — Summary of mapping statistics. (PDF 22 kb) [file 12890_2017_400_MOESM1_ESM.pdf]

Table S1. Summary of mapping statistics

Family ID	SampleID	Disease status	Application	Number of read		Number of bases		Precent		Number of variants[b]	
				Total	Mapped	Total	Mapped	Mapped reads	Target covered[a]	SNVs	Indels
1	OM0054	Proband	Whole Exome	104,766,655	77,459,744	7,857,499,125	5,809,480,800	73.9	87.4	36,692	3,288
2	OM0070	Proband	Whole Exome	61,430,098	46,182,581	4,607,257,350	3,463,693,575	75.2	75.3	36,575	2,869
2	OM0071	Affected	Whole Exome	67,237,382	51,396,054	5,042,803,650	3,854,704,050	76.4	78.1	36,503	2,897
3	OM0095	Proband	Whole Exome	86,583,264	66,575,106	6,493,744,800	4,993,132,950	76.9	83.4	36,803	3,110
4	OF0001	Unaffected	Whole genome	847,462,500	734,821,441	127,966,837,500	110,958,037,591	86.7	99.1	37,421	3,426
4	OF0002	Unaffected	Whole genome	885,665,298	788,335,262	133,735,459,998	119,038,624,562	89.0	99.5	38,363	3,542
4	OF0003	Unaffected	Whole genome	872,840,784	777,165,010	131,798,958,384	117,351,916,510	89.0	99.3	38,201	3,542
4	OM0106	Proband	Whole genome	826,178,940	748,033,875	124,753,019,940	112,953,115,125	90.5	99.5	38,397	3,526
5	OM0079	Proband	Whole Exome	80,932,581	56,232,920	6,069,943,575	4,217,469,000	69.5	81.0	36,276	3,048
5	OM0195	Affected	Whole Exome	77,597,032	53,761,375	5,819,777,400	4,032,103,125	69.3	77.9	36,058	2,813
6	OM0104	Proband	Whole Exome	74,455,210	56,899,050	5,584,140,750	4,267,428,750	76.4	81.3	37,315	3,053
6	OM0178	Affected	Whole Exome	60,606,131	42,458,335	4,545,459,825	3,184,375,125	70.1	72.5	35,438	2,733
7	OM0065	Proband	Whole genome	656,095,930	606,786,532	99,070,485,430	91,624,766,332	92.5	99.2	38,590	3,620
8	OM0188	Proband	Whole genome	821,829,322	765,808,847	124,096,227,622	115,637,135,897	93.2	99.6	38,434	3,479
9	OM0250	Proband	Whole genome	779,018,328	721,561,175	117,631,767,528	108,955,737,425	92.6	99.5	38,625	3,486
9	OM0251	Unaffected	Whole genome	752,035,468	692,702,678	113,557,355,668	104,598,104,378	92.1	99.1	38,477	3,440
9	OM0252	Unaffected	Whole genome	800,743,792	746,311,548	120,912,312,592	112,693,043,748	93.2	99.6	38,309	3,423

[a] Percent of total targeted bases covered at least 10x depths.

[b] Number and type of variants on conding regions.
