# Supplementary figures and images for: A burden of rare variants in BMPR2 and KCNK3 contributes to a risk of familial pulmonary arterial hypertension
Source: BMC Pulm Med. 2017 Apr 7;17:57. doi: 10.1186/s12890-017-0400-z (PMC5383973; doi:10.1186/s12890-017-0400-z)

Figure S1

a

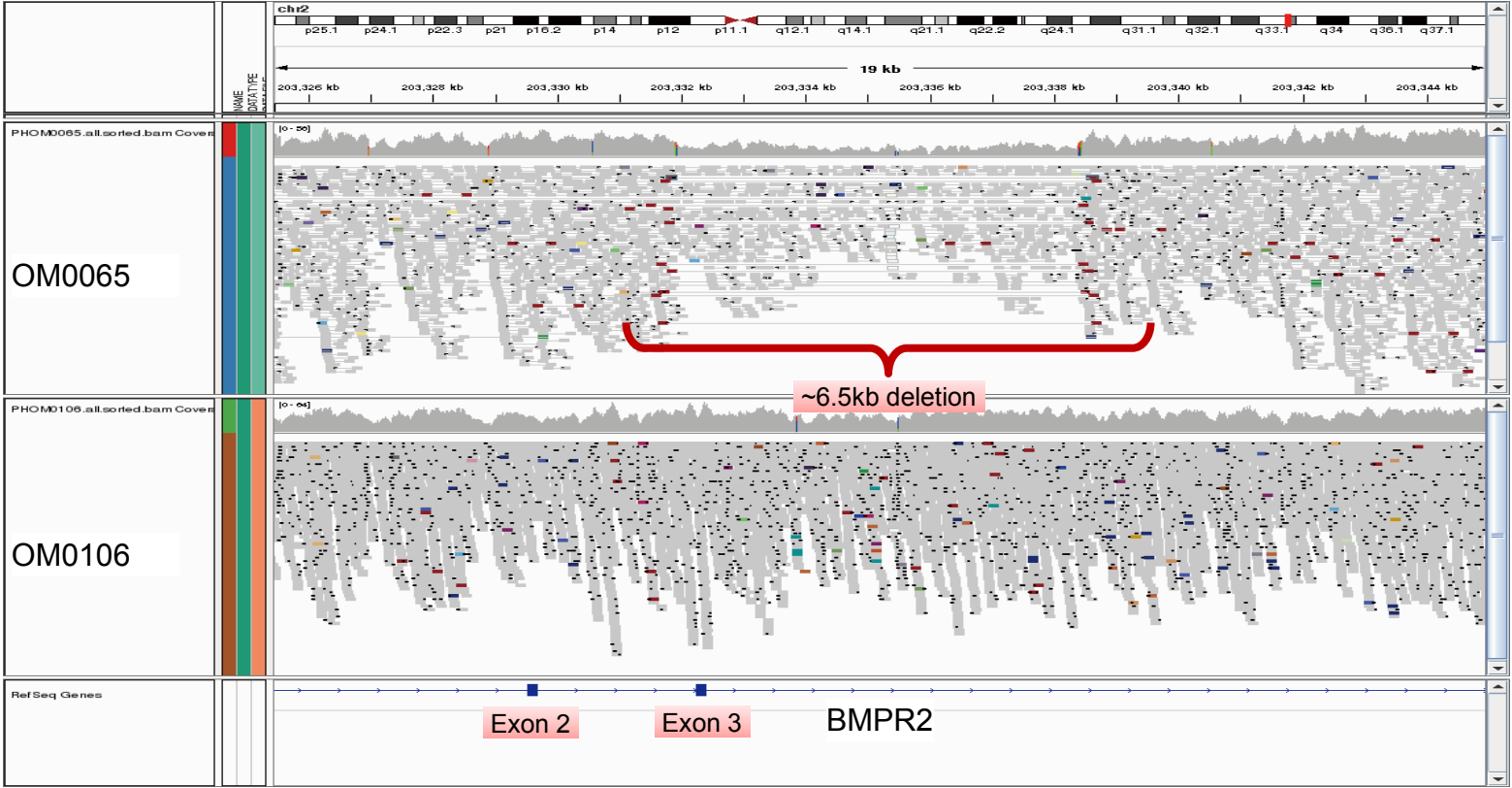

b

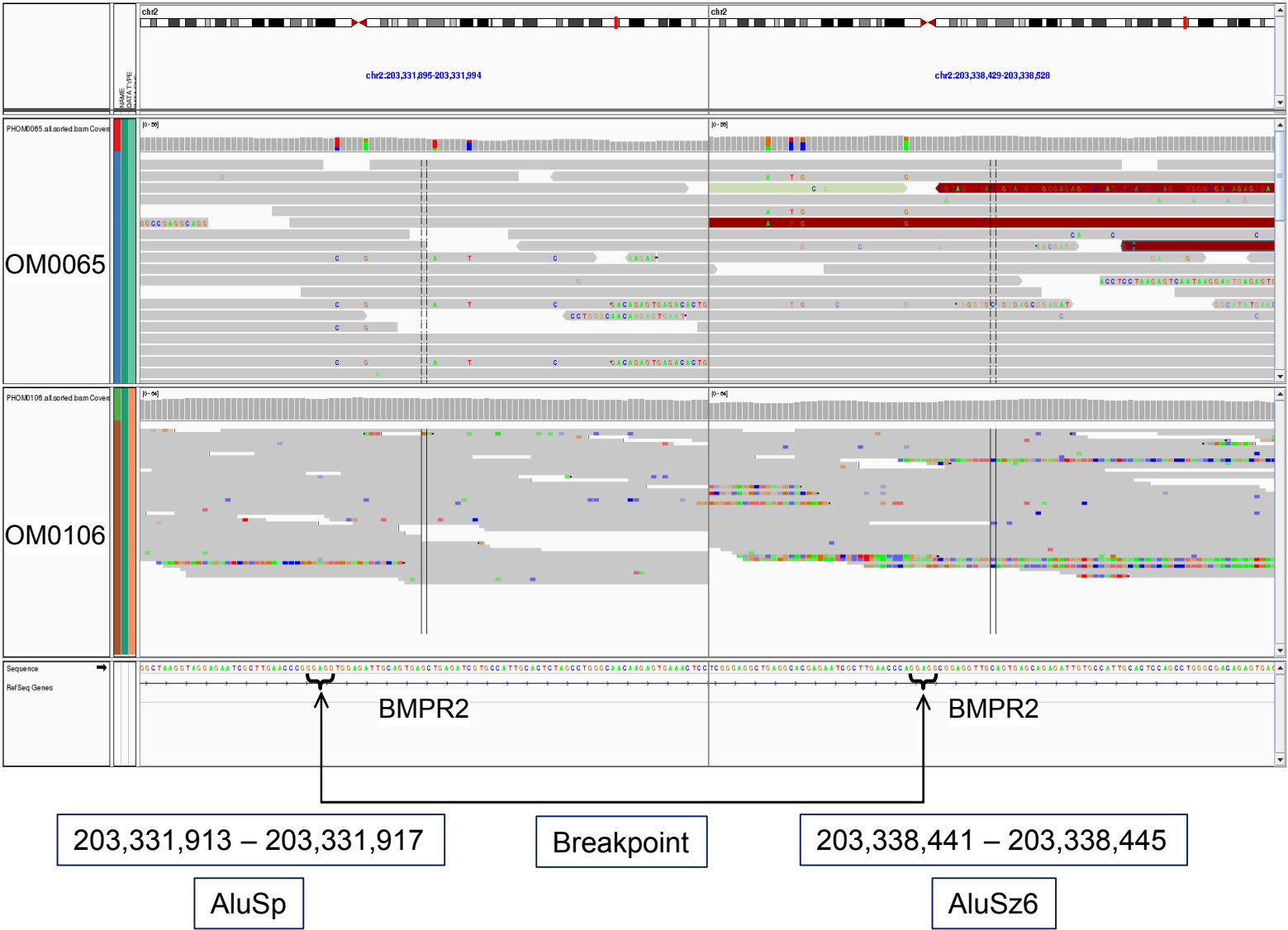

Supplement: Supplementary file 2 — Large deletion found in the patient in family 8. (a) The large deletion was supported by depth of coverage and discordant paired-end reads. (b) The length of the deletion was approximately 6.5 kilobases and spanned the entire region of exon 3. Breakpoints of the large heterozygous deletion were located in the Alu repeat sequences, which were supported by read depths and paired-end reads spanning the long region. (PDF 529 kb) [file 12890_2017_400_MOESM2_ESM.pdf]

Figure S2

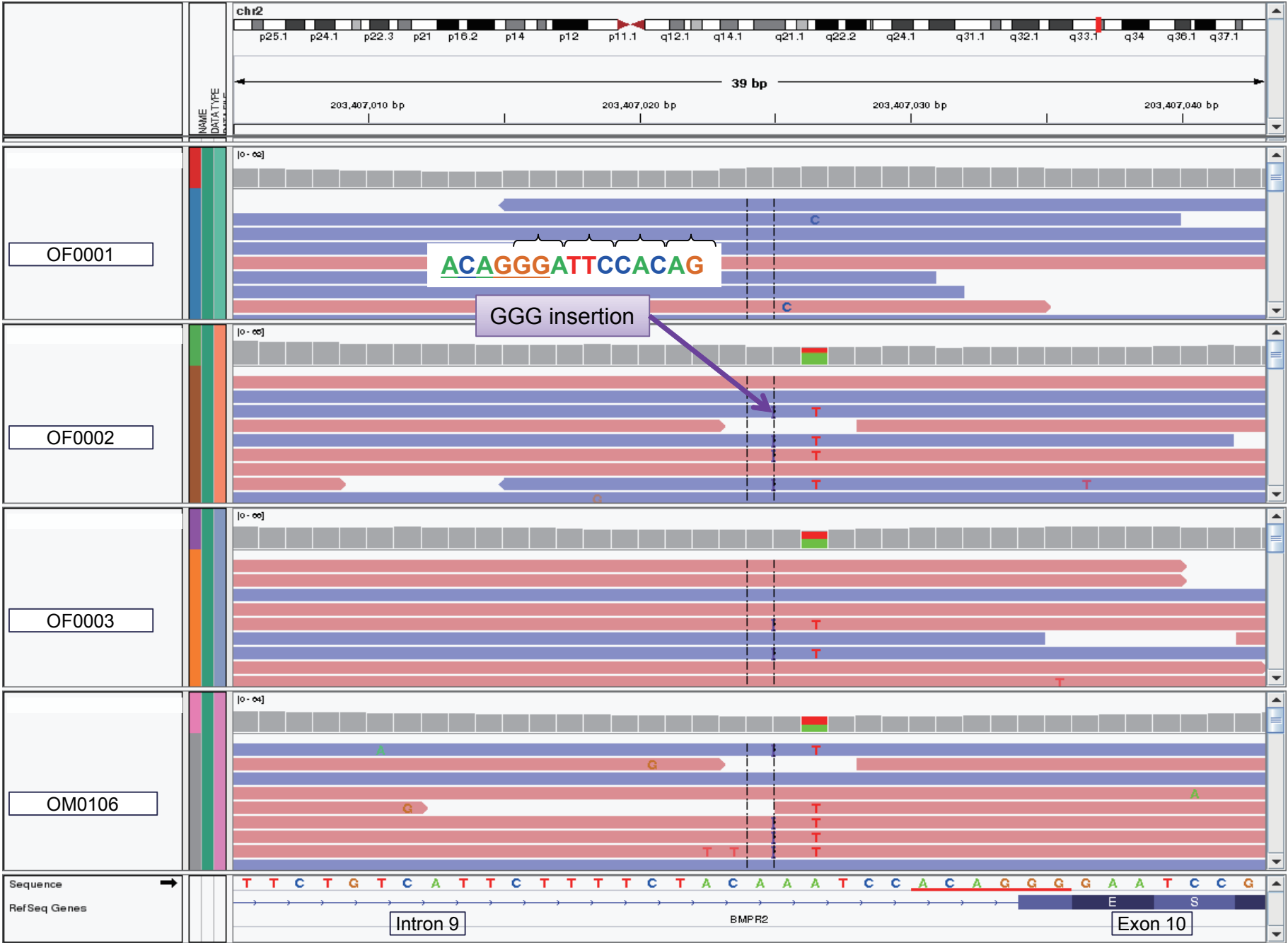

Supplement: Supplementary file 3 — Possible pathogenic variants found in the intron of BMPR2 in family 4. A rare three base insertion (GGG) at 10 bases upstream from exon 10 creates identical sequences around the canonical splicing site (ACAGGG). By the insertion, an out-of-frame protein could be translated due to the new splice acceptor site. (PDF 411 kb) [file 12890_2017_400_MOESM3_ESM.pdf]

Figure S4

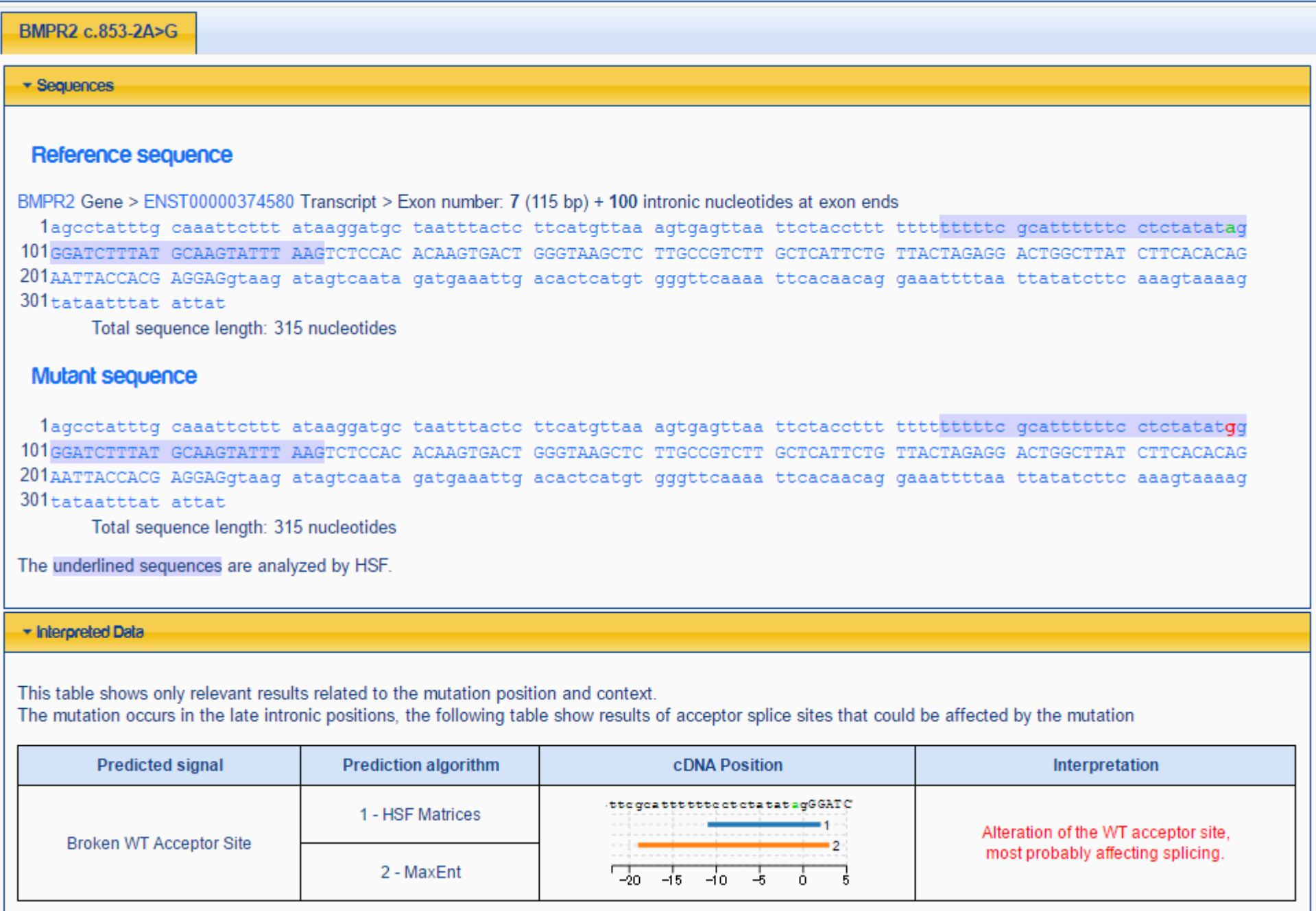

Supplement: Supplementary file 5 — Disruption of wild type acceptor site by the single nucleotide variants (c.853-2A > G) predicted from in silico programs. (PDF 184 kb) [file 12890_2017_400_MOESM5_ESM.pdf]
