# Supplementary material for: A burden of rare variants in BMPR2 and KCNK3 contributes to a risk of familial pulmonary arterial hypertension
Source: BMC Pulm Med. 2017 Apr 7;17:57. doi: 10.1186/s12890-017-0400-z (PMC5383973; doi:10.1186/s12890-017-0400-z)
Supplement: Supplementary file 4 — Creation of the new splice acceptor site by the three base insertion (NM_001204.6:c.1277-10_1277-9insGGG) predicted from in silico programs. (PDF 218 kb) [file 12890_2017_400_MOESM4_ESM.pdf]

Figure S3

BMPR2 c.1277-10_1277-9insGGG

Sequences

Reference sequence

BMPR2 Gene > ENST00000374580 Transcript > Exon number: **10** (137 bp) + **100** intronic nucleotides at exon ends

```
  1 caattttttt tgcttacttg gtatcagaaa taccсctgtt attctatcat ttatgatagt tagaaatttt attctgtcat tcttttctac a---aatcca
101 cagGGGAATC CGTACCAGAG TACCAGATGG CTTTTCAGAC AGAGGTTGGA AACCATCCCA CTTTTGAGGA TATGCAGGTT CTCGTGTCTA GGGAAAAACA
201 GAGACCCAAG TTCCCAGAAG CCTGGAAAGA AAATAGCCTG gtaagaaaaa actaagttat taaagagagg acttatgact gaatatgttt taatgttgtt
301 tgaaacaaaa atagactgtt aataacgtta atagtttttg
```

Total sequence length: 340 nucleotides

Mutant sequence

```
  1 caattttttt tgcttacttg gtatcagaaa taccсctgtt attctatcat ttatgatagt tagaaatttt attctgtcat tcttttctac agggaatcca
101 cagGGGAATC CGTACCAGAG TACCAGATGG CTTTTCAGAC AGAGGTTGGA AACCATCCCA CTTTTGAGGA TATGCAGGTT CTCGTGTCTA GGGAAAAACA
201 GAGACCCAAG TTCCCAGAAG CCTGGAAAGA AAATAGCCTG gtaagaaaaa actaagttat taaagagagg acttatgact gaatatgttt taatgttgtt
301 tgaaacaaaa atagactgtt aataacgtta atagtttttg
```

Total sequence length: 340 nucleotides

The underlined sequences are analyzed by HSF.

Interpreted Data

This table shows only relevant results related to the mutation position and context.
The mutation occurs in the late intronic positions, the following table show results of acceptor splice sites that could be affected by the mutation

<table>
<tr><th>Predicted signal</th><th>Prediction algorithm</th><th>cDNA Position</th><th>Interpretation</th></tr>
<tr><td rowspan="2">New Acceptor Site</td><td>1 - HSF Matrices</td><td rowspan="2">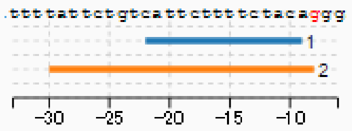</td><td rowspan="2">Activation of an intronic cryptic acceptor site.
Potential alteration of splicing.</td></tr>
<tr><td>2 - MaxEnt</td></tr>
<tr><td>New Acceptor Site</td><td>1 - HSF Matrices</td><td>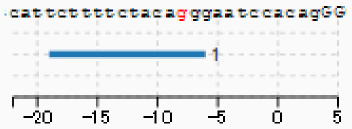</td><td>Activation of an intronic cryptic acceptor site.
Potential alteration of splicing.</td></tr>
<tr><td>Broken WT Acceptor Site</td><td>1 - MaxEnt</td><td>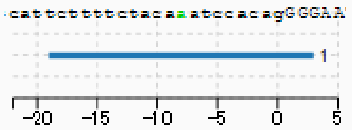</td><td>Alteration of the WT acceptor site,
most probably affecting splicing.</td></tr>
</table>
